# 

**Published:** 1908-04-25

**Authors:** 


CONTENTS.

| | PAGE |
|---|---|
| **Disorders of the Heart** ... | 81 |
| **The Predominance of the Clinician** ... | 82 |
| **Annotations**— | |
| Beneficent Bacteria—The Infants Hospital—Slaughter House Reform ... | 83 |
| **Medical Opinion and Movement** ... | 84 |
| **Hospital Clinics**— | |
| A Case of Cancer of the Liver. By Wm. Hale White, M.D. Lond., F.R.C.P. ... | 87 |
| **Special Article**— | |
| The Ophthalmo-Tuberculin Reaction in Private Practice ... | 90 |
| **Medicine**— | |
| Early Tuberculosis of the Kidney ... | 91 |
| **Practical Notes on Diagnosis and Treatment** ... | 92 |
| **Points in Surgery**— | |
| Aneurysm ... | 93 |
| Some Aspects of General Peritonitis—III. ... | 94 |
| **Obstetrics**— | |
| Pregnancy Complicated by Ovarian Tumours ... | 95 |
| **Dermatology**— | |
| Acne Vulgaris ... | 96 |
| **Laryngology and Rhinology**— | |
| Functional Aphonia ... | 97 |
| **Therapeutics**— | |
| Prescriptions and Dispensing ... | 98 |
| **Medico-Legal Points**— | |
| Manslaughter by Administering Noxious Drugs ... | 99 |
| **Resident Medical Officers' Department**— | |
| Difficulty in Micturition in Nervous Patients Admitted to Hospital Wards ... | 100 |
| **The General Practitioner's Column**— | |
| The Personality of the Physician ... | 101 |
| **New Appliances and Things Medical**— ... | 102 |
| **Hospital Administration** | |
| Graduate Study on the Continent.—Germany ... | 103 |
| Social and Poor-law Problems ... | 104 |
| News and Coming Events ... | 105 |
| **Nursing Administration**— | |
| Queen Victoria's Jubilee Institute for Nurses ... | 107 |
| **Editor's Letter-Box**— | |
| Australia for the Sons of Professional Men ... | 108 |

